# Endophytic *Trichoderma* species associated with *Theobroma cacao* L.: identification and biocontrol potential against *Moniliophthora roreri* and *Fusarium* sp.

**DOI:** 10.3389/fmicb.2026.1811538

**Published:** 2026-05-15

**Authors:** Henry W. Santillan-Culquimboz, Vilma Aguilar-Rafael, Jorge Ronny Díaz-Valderrama, Flavio Lozano-Isla, Enistein R. Reyna-Rivera, Santos T. Leiva-Espinoza

**Affiliations:** 1 Instituto de Investigación Para el Desarrollo Sustentable de Ceja de Selva (INDES-CES), Universidad Nacional Toribio Rodríguez de Mendoza de Amazonas, Chachapoyas, Peru; 2 Facultad de Ingeniería y Ciencias Agrarias, Universidad Nacional Toribio Rodríguez de Mendoza de Amazonas (UNTRM), Chachapoyas, Peru; 3 Grupo de Investigación en Fitopatología y Micología, Instituto de Investigación Para el Desarrollo Sustentable de Ceja de Selva (INDES-CES), Universidad Nacional Toribio Rodríguez de Mendoza de Amazonas, Chachapoyas, Peru; 4 Grupo de Investigación en Procesos en Sanidad Vegetal, Instituto de Investigación Para el Desarrollo Sustentable de Ceja de Selva (INDES-CES), Universidad Nacional Toribio Rodríguez de Mendoza de Amazonas, Chachapoyas, Peru; 5 Centro de Investigación e Innovación en Granos y Semillas, Universidad Nacional Toribio Rodríguez de Mendoza de Amazonas (UNTRM), Chachapoyas, Peru

**Keywords:** antagonism, antibiosis, biological control, endophytes, molecular identification, mycoparasitism, sustainability

## Abstract

**Introduction:**

Peru, recognized as part of the center of origin of cacao, harbors multiple varieties highly valued for their unique characteristics and sensory profile. However, phytosanitary constraints threaten its sustainability, genetic diversity, and productivity.

**Methods:**

This study aimed to identify endophytic *Trichoderma* strains with biocontrol potential against Moniliophthora roreri and *Fusarium* sp. A multilocus analysis (ITS, *tef1-α*, and *rpb2*) was performed, complemented by morphological characterization. *In vitro* antagonistic capacity was evaluated through antibiosis, mycoparasitism, and antagonism assays.

**Results:**

Phylogenetic and morphological analyses allowed the identification of *Trichoderma afroharzianum*, *T. parareesei*, *T. sexdentis*, *T. strigosum*, *T. reesei*, and *T. erinaceum*. Additionally, several isolates could not be assigned to known taxa, suggesting the presence of potential new species. This study constitutes the first report of *T. sexdentis* and *T. strigosum* as endophytes associated with cacao. Antagonism assays, based on antibiosis and mycoparasitism mechanisms, demonstrated that the strains exhibit high antagonistic potential against *M. roreri* (approximately 80%) and *Fusarium* sp. (approximately 70%).

**Discussion:**

These findings underscore the limited characterization of endophytic fungal communities in Peruvian cacao systems and highlight the ecological and biotechnological relevance of *Trichoderma* as promising biocontrol agents for the sustainable management of cacao pathogens.

## 1 Introduction

The socioeconomic impact of cacao (*Theobroma cacao*) has been key to the development of producing regions worldwide (Santillan-Culquimboz et al., 2025). It is estimated that between 40 and 50 million people depend directly or indirectly on its cultivation as a source of livelihood (Beg et al., 2017). This dependence is based on a model dominated by smallholder farmers, who produce around 90% of the world’s cocoa on plots of 2 to 5 hectares and with low levels of technification (Beg et al., 2017; Kongor et al., 2024). This system presents a yield gap of 80–95% relative to the technical potential, largely due to disease pressure and the dependence on agrochemicals, which compromise the profitability and stability of agroecosystems (Kongor et al., 2024). The urgency of implementing sustainable strategies is manifested more intensely in the territories that sustain the global supply.

Cocoa production is concentrated mainly in developing countries (Santillan-Culquimboz et al., 2025). West Africa constitutes the principal producing region, contributing around 70% of the global volume, with Côte d’Ivoire and Ghana as production leaders (Ariza-Salamanca et al., 2023; Kongor et al., 2024; FAOSTAT, 2024). Latin America accounts for approximately 20% of total production, with countries such as Brazil, Ecuador, and Peru standing out, where fine-flavor cocoa holds increasing strategic value within specialized markets (Charry et al., 2025; FAOSTAT, 2024). Peru is a key player and a leader in global organic cocoa production, recognized for its focus on quality and sustainability (Rojas-Briceño et al., 2022; Gurbillón et al., 2025).

Peru, recognized as a fundamental part of the center of origin and primary genetic diversity of cocoa, harbors approximately 60% of the genotypes existing worldwide (Díaz-Valderrama et al., 2020; Gutierrez et al., 2022). This reservoir is reflected in three native cultivars of fine-flavor cocoa with high commercial value: ‘Chuncho’, ‘Cacao Amazonas Perú’ (which has a protected designation of origin; INDECOPI, 2016), and ‘Blanco Piurano’ (Díaz-Valderrama et al., 2020). These germplasms exhibit distinctive organoleptic profiles and a genetic background closely linked to the Criollo and Nacional groups, consolidating them as strategic resources for specialized markets (Díaz-Valderrama et al., 2020; Cabrera-De-La-Cruz et al., 2024). However, the sustainability of this strategic resource is compromised by the presence of important phytosanitary constraints that affect the crop from the initial nursery stages to the productive phase in the field, with negative impacts on productivity, the conservation of genetic diversity, and the stability of cocoa-based systems (Leiva et al., 2022).

Among these threats, *Moniliophthora roreri*, the causal agent of frosty pod rot, stands out as one of the most destructive diseases (Rodríguez-Velázquez et al., 2024; Llanos-Gómez et al., 2026). Its high invasive potential, diversity, and adaptability allow it to colonize entire plantations under favorable conditions, leading to the abandonment of vast cultivation areas (Ploetz, 2016; Leiva et al., 2022). Complementarily, species of the *Fusarium* genus have been associated with vascular wilt and seedling mortality in nurseries, with their aggressiveness being modulated by environmental factors such as relative humidity and temperature (Rosmana et al., 2013; Mulatu et al., 2023). These limitations have driven the search for environmentally friendly alternatives to overcome the problems caused by traditional chemical control methods (Leiva et al., 2022).

The use of natural enemies such as *Trichoderma* spp. has proven to be a sustainable alternative in cacao production systems (Leiva et al., 2022). This fungus exerts antagonism through direct and indirect mechanisms, including mycoparasitism, antibiosis, and competition for nutrients and space (Santillan-Culquimboz et al., 2025). Furthermore, it has the ability to colonize plant tissues as an endophyte, providing its host plant with various types of induced resistance: systemic acquired resistance (SAR) and induced systemic resistance (ISR), enhancing defense against pathogen attacks (Santillan-Culquimboz et al., 2025; Yang et al., 2025). Therefore, identifying *Trichoderma* strains capable of establishing effective antagonistic and endophytic interactions is essential to support their rational and efficient use in biological control programs for cacao.

The delimitation of species within the genus *Trichoderma* presents a taxonomic challenge due to the similarity of its phenotypic traits and the cryptic nature of its morphological characters (Brito et al., 2023). In light of this limitation, molecular identification through multilocus analysis is currently the most accurate methodology for species discrimination within this genus (Zhao et al., 2023). Therefore, the main objective of this study is to identify endophytic *Trichoderma* strains with biological control potential against *Moniliophthora roreri* and *Fusarium* sp. To achieve this, the following specific objectives are set: (i) molecularly identify endophytic *Trichoderma* strains isolated from fine-flavor native cacao plants; (ii) characterize these strains morphologically; and (iii) assess their biological control potential.

## 2 Methodology

### 2.1 Study area

**Figure 1:**
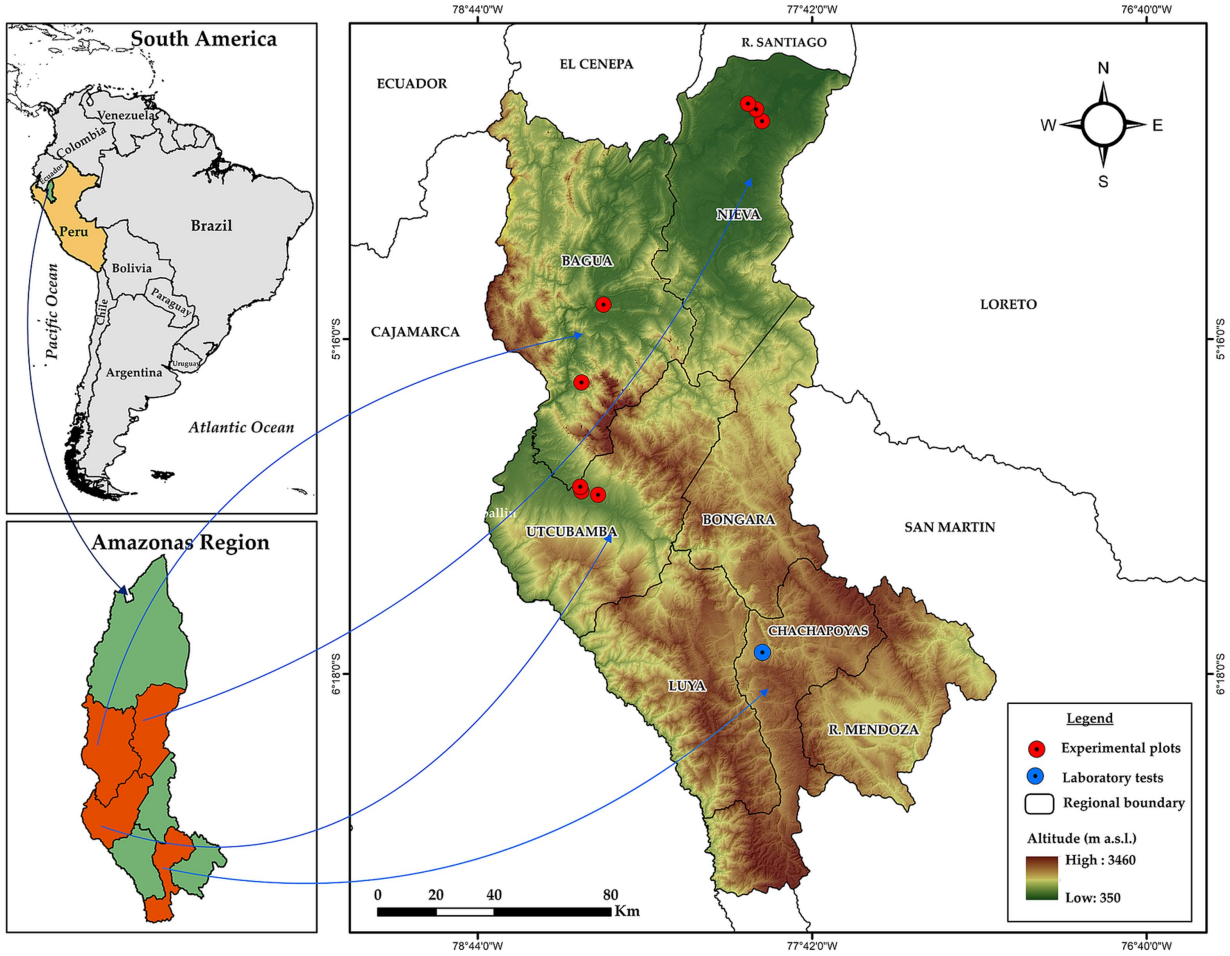
Location map of the study sites in the Amazonas region, Peru. Red points indicate the sampling sites in the provinces of Condorcanqui (Nieva district), Bagua, and Utcubamba for the isolation of *Trichoderma*. The blue point represents LABISANV-UNTRM in Chachapoyas, where the phytopathogens were obtained and the samples were processed.

The present study involved of field and laboratory activities. The sample collection was carried out in the provinces of Condorcanqui, Bagua, and Utcubamba, located in the Amazonas region, Peru. The research was conducted at the Plant Health Research Laboratory (LABISANV) of the Institute for Sustainable Development of Ceja de Selva (INDES-CES), Toribio Rodríguez de Mendoza National University of Amazonas (UNTRM) (Figure 1).

### 2.2 Isolation of *Trichoderma* endophytes

Cacao plants (*Theobroma cacao* L.) of the Fine Flavor type were selected according to previously established criteria: absence of visible disease symptoms, adequate nutritional and physiological status, apparent general vigor, and no prior exposure to any type of chemical or biological product application (Hanada et al., 2010). Samples of vascular tissue (xylem) from the main trunk, as well as segments of fine roots (< 5 mm in diameter) and secondary branches, were collected from each plant.

Prior to sampling, the stem surface was disinfected with 70% ethanol. Using a sterile scalpel, previously flamed, a section of the bark approximately 16 cm^2^ was removed, from which four fragments of vascular tissue, each 25 mm^2^, were obtained. The fragments were immediately placed on Petri dishes containing Potato Dextrose Agar (PDA) medium supplemented with chloramphenicol (25 μg/mL) to inhibit bacterial growth (Samuels et al., 2006; Hanada et al., 2008; Mejía et al., 2008).

Root and branch samples were transported to the laboratory in an isothermal container with refrigerants, maintaining a temperature between 4 and 8 °C to preserve their physiological and microbiological integrity (Arnold and Herre, 2003). All material was processed within 6 hrs after collection. Initially, the tissues were washed with running water to remove soil debris and superficial impurities. Subsequently, under aseptic conditions in a laminar flow cabinet, surface disinfection was performed by immersion in 70% alcohol for 30 s, followed by treatment with 1.5% sodium hypochlorite for 2 min and two consecutive rinses with sterile distilled water.

After disinfection, a superficial layer of approximately 1 mm was removed from the tissue using a sterile scalpel. The isolation of fragments from the intact internal region followed the same procedure described previously. All plates were incubated at 25 ± 1 °C in continuous darkness, following the protocol described by Hanada et al. (2008).

### 2.3 Isolation of plant pathogens

Reference isolates of *Moniliophthora roreri*, the causal agent of frosty pod rot in cacao, and *Fusarium* sp., were obtained from the mycological culture collection of the Plant Health Research Laboratory (LABISANV) at the Institute for Sustainable Development of Ceja de Selva (INDES-CES), Toribio Rodríguez de Mendoza National University of Amazonas (Leiva et al., 2022).

### 2.4 Molecular phylogenetic analysis

Genomic DNA extraction was performed from approximately 50 mg of fresh mycelium, obtained by scraping pure *Trichoderma* colonies with 48–72 h of growth (Jiang et al., 2016). The procedure was carried out using the commercial Wizard® Genomic DNA Purification kit (Promega Corporation, Madison, WI, United States), following the manufacturer’s instructions with adaptations for filamentous fungi.

The ITS ribosomal region and the coding genes tef1-*α* and rpb2 were amplified using polymerase chain reaction (PCR). The reactions were prepared in a final volume of 25 μL, consisting of 12.5 μL of Master Mix (Promega, Madison, WI, United States), 1.25 μL of each primer (10 μM), 5 μL of genomic DNA, and 5 μL of nuclease-free water. For the amplification of the ITS region, primers ITS1F and ITS4 were used (White et al., 1990; Haque et al., 2020); for the *tef1-α* gene, primers EF1 and EF2 were used (O’Donnell et al., 1998); and for the *rpb2* gene, primers fRPB2-5F and fRPB2-7cR were used (Liu et al., 1999).

The sequencing of the PCR products was performed by Macrogen (Santiago, Chile) using the Sanger method. Consensus sequences for each marker were generated from the obtained chromatograms through assembly and manual editing using Sequencher v.5.4 software. The preliminary taxonomic identity of the sequences was assessed by similarity searches in the GenBank database of NCBI, using the BLASTN algorithm (Altschul et al., 1990; Zhao et al., 2023).

Based on the results of the preliminary identification, representative sequences of the phylogenetically closest species available in public databases were selected. For each species, sequences of reference strains (holotype, ex-type, or valid epitype) were also included (Table 1), according to the taxonomic protocol proposed by Cai and Druzhinina (2021). Multiple sequence alignments were performed using the MUSCLE algorithm, implemented in MEGA v.12 software (Kumar et al., 2024). Subsequently, the alignments were manually edited at the 5′ and 3′ ends to retain only informative and high-quality regions.

**Table 1:** Strain numbers and GenBank accession numbers corresponding to the sequences used for the phylogenetic analyses.

Species	Strain number	ITS	*rpb2*	*tef1-α*
*Protocrea farinosa*	CBS 121551^T^	–	EU703935	EU703889
*Protocrea pallida*	CBS 299.78^T^	EU703925	EU703948	EU703900
*Trichoderma afarasin*	DIS 314F	FJ442259	FJ442778	FJ463400
*T. afarasin*	DIS 377a^T^	–	FJ442799	FJ463322
***T.* sp.**	**HWSC10**	**PX990075**	**PZ053742**	**PZ273066**
***T.* sp.**	**HWSC11**	**PX990076**	**PZ053743**	**PZ273067**
***T.* sp.**	**HWSC12**	**PX990077**	**PZ053744**	**PZ273068**
*T. afroharzianum*	CBS 124620^ET^	FJ442265	FJ442691	FJ463301
***T. afroharzianum***	**HWSC06**	**PX990073**	**PZ053740**	**PZ273052**
***T. afroharzianum***	**HWSC07**	**PX990074**	**PZ053741**	**PZ273053**
*T. albofulvopsis*	9930^T^	–	KU529138	KU529127
*T. atrobrunneum*	G. J. S. 05–101	FJ442677	FJ442745	FJ463392
*T. atroviride*	CBS 142.95^ET^	–	EU341801	AF456891
*T. azevedoi*	CEN1422^T^	–	MK696821	MK696660
*T. bannaense*	HMAS248840^T^	KY687923	KY687979	KY688037
*T. citrinoviride*	DAOM 172792^T^	EU280098	KJ842210	KJ713208
*T. dorotheae*	GJS 99-202^T^	NR_166014	EU248602	DQ307536
*T. dorothopsis*	HZA5^ET^	–	MH647795	MK850827
*T. effusum*	C. P. K.254 = DAOM230007^T^	DQ083008	KJ665260	KJ665473
*T. erinaceum*	DAOM 230019^T^	DQ083009	KJ842151	AY750880
***T. erinaceum***	**HWSC27**	**PX990084**	**PZ053751**	**PZ273060**
*T. euskadiense*	S377 = CBS130013^T^	–	KJ665269	KJ665492
*T. ghanense*	G. J. S.95–137 = IAM13109^T^	NR_120299	JN175559	AY937423
*T. gracile*	CBS130714 = G. J. S.10263^T^	–	JN175547	JN175598
*T. graminis*	YNE00410 = GDMCC 3.1013^T^	–	OR779491	OR779514
*T. guizhouense*	CBS131803^T^	JN191311	JQ901400	JN215484
*T. harzianum*	CBS226.95^T^	AY605713	AF545549	AF348101
*T. inhamatum*	CBS273.78^T^	FJ442680	FJ442725	AF348099
*T. istrianum*	CBS 130539^T^	–	KJ665281	KJ665523
*T. koningii*	7723^T^	KJ783285	KJ634720	KJ634753
*T. koningiopsis*	GJS 93–20^T^	DQ313140	EU241506	DQ284966
*T. lixii*	CBS110080 = GJS97-96	AF443920	KJ665290	FJ716622
*T. longibrachiatum*	TUFC61535 = CBS816.68^T^	EU401556	DQ087242	EU401591
*T. merleae*	MST FP3586^T^	PQ570877	PQ572711	PQ572712
*T. ovalisporum*	DIS 70a = CBS 113299^T^	AY380897	FJ442742	AY376037
*T. parareesei*	CBS125925 = C. P. K. 717 = TUBF–1066^T^	NR_138453	HM182963	GQ354353
***T. parareesei***	**HWSC13**	**PX990078**	**PZ053745**	**PZ273054**
***T. parareesei***	**HWSC16**	**PX990079**	**PZ053746**	**PZ273055**
*T. pseudoasiaticum*	YMF 1.6178^T^	–	MT052183	MT070155
*T. pyramidale*	S73 = CB135574^T^	–	KJ665334	KJ665699
*T. reesei*	DAOM 167654^T^	NR_120297	KJ842213	KJ713193
*T. reesei*	QM6a = CBS383.78^T^	MH861154	HM182969	Z23012
***T. reesei***	**HWSC26**	**PX990083**	**PZ053750**	**PZ273059**
*T. rifaii*	CBS130745	FJ442621	FJ442720	FJ463321
*T. saturnisporum*	ATCC18903 = CBS 330.70 = C. P. K.1266^T^	NR_103704	DQ087243	EU280044
*T. sexdentis*	URMICRO 11733^T^	–	PQ334915	PQ334916
***T. sexdentis***	**HWSC18**	**PX990080**	**PZ053747**	**PZ273056**
***T. sexdentis***	**HWSC21**	**PX990082**	**PZ053749**	**PZ273057**
***T.cf. sexdentis***	**HWSC01**	**PX990068**	**PZ053735**	**PZ273061**
***T.cf. sexdentis***	**HWSC02**	**PX990069**	**PZ053736**	**PZ273062**
***T.cf. sexdentis***	**HWSC03**	**PX990070**	**PZ053737**	**PZ273063**
***T.cf. sexdentis***	**HWSC04**	**PX990071**	**PZ053738**	**PZ273064**
***T.cf. sexdentis***	**HWSC05**	**PX990072**	**PZ053739**	**PZ273065**
*T. shaanxiensis*	T32000 = GDMCC 3.1014^T^	–	OR779486	OR779513
*T. sinense*	DAOM230004	NR_134425	JN175528	KJ713191
*T. songyi*	SFC20130926-S001	MG491505	KJ636518	KJ636525
*T. strigosellum*	G. J. S. 05-02^T^	EU263997	EU248607	EU248631
*T. strigosum*	DAOM 166121^T^	EU280120	AF545556	EU280019
***T. strigosum***	**HWSC19**	**PX990081**	**PZ053748**	**PZ273058**
*T. texanum*	LESF551^T^	HQ608136	KT278920	KT278988
*T. tibetica*	YMF 1.05583^T^	MK779177	MK779178	MK779179
*T. viride*	CBS 119325^ET^	NR_138441	EU711362	DQ672615
*T. zelobreve*	CGMCC 3.19695^T^	MN594474	MN605872	MN605883

The sequences from this study are highlighted in bold. T indicates a type culture. ET indicates an epitype culture.

Phylogenetic analyses were performed using the Maximum Likelihood (ML) method, both for each individual locus (*tef1-α* and *rpb2*) and for a concatenated dataset that included all the analyzed markers (Cai and Druzhinina, 2021). The analyses were executed on the CIPRES Science Gateway platform, using the RAxML-HPC BlackBox v.8.2.12 tool and the GTRGAMMA substitution model (Stamatakis, 2014). Visualization and editing of the phylogenetic trees were carried out using FigTree v.1.4.3 software. Branches that presented bootstrap support values under ML greater than 70% were considered statistically well-supported (Zhao et al., 2023).

### 2.5 Morphological characterization

Morphological characterization was carried out as a complement to molecular identification. For macroscopic evaluation, the isolates were cultured on Potato Dextrose Agar (PDA) and Synthetic Nutrient Agar (SNA) media and incubated for 10 days at 25 ± 1 °C. During this period, cultural characteristics such as colony color, mycelial texture, growth pattern, and pigment production were recorded.

Microscopic characterization was performed using the microculture technique on SNA, selected for its ability to induce the formation and differentiation of reproductive structures typical of the genus *Trichoderma* (Nascimento Brito et al., 2023). The microscopic preparations were mounted in lactoglycerol and stained with lactophenol blue to facilitate the observation of the fungal structures.

The observation and photographic documentation were performed using an Olympus BX53 optical microscope, coupled with an Olympus DP74 digital camera. Image capture and the morphometric analysis of conidia and conidiophores were conducted using OLYMPUS cellSens Dimension software, following the manufacturer’s technical specifications.

### 2.6 Biological control assays

The biological control capacity of endophytic *Trichoderma* strains was evaluated by quantifying two of their main mechanisms of action: antibiosis and mycoparasitism. Based on the results obtained, the antagonistic potential of the strains was determined. All assays were conducted under controlled conditions of photoperiod (12 h light/12 h darkness) and a temperature of 25 ± 1 °C, with five replications per treatment in each experiment.

#### 2.6.1 Antibiosis assays

The inhibitory potential of endophytic *Trichoderma* strains against cacao-associated plant pathogens was evaluated through antibiosis assays, using the dual confrontation or pairwise comparison method, as described by Holmes et al. (2004). For this, 5 mm diameter mycelial discs were obtained from 12-day-old cultures of *Moniliophthora roreri* and *Fusarium* sp. Each disk was placed at the periphery of a 90 mm diameter Petri dish containing Potato Dextrose Agar (PDA). The plant pathogens were incubated for 6 days to allow for their initial establishment.

After this period, a mycelial disk of *Trichoderma* (5 mm in diameter) was inoculated at the opposite end of the plate, directly facing the growth of the plant pathogen. Antagonistic interactions were monitored every 24 h until physical contact between both mycelia was observed. As controls, plates with individual growth of each plant pathogen in the absence of *Trichoderma* were included.

The average time until mycelial contact was 3 days for *M. roreri* and 4 days for *Fusarium* sp. At this point, measurements were taken to quantify the percentage of radial growth inhibition using the formula proposed by Holmes et al. (2004):

PA=[(RG−RGT)/RG]×100

Where PA corresponds to the percentage of antibiosis, RG to the radial growth of the plant pathogen on control plates (mm), and RGT to the radial growth of the plant pathogen in the presence of *Trichoderma* (mm).

#### 2.6.2 Mycoparasitism assays

The mycoparasitism potential of *Trichoderma* strains against the evaluated plant pathogens was determined using the pre-colonized plate method, as described by Bailey et al. (2008) and Leiva et al. (2022). Initially, mycelial discs of *Moniliophthora roreri* and *Fusarium* sp., from 12-day-old cultures, were inoculated at the periphery of 90 mm diameter Petri dishes containing Potato Dextrose Agar (PDA) medium. The plates were incubated until the plant pathogens completely colonized the medium (100% coverage).

Once the pathogen had completely colonized the medium, a mycelial strip of *Trichoderma* approximately 2.5 × 0.5 cm, obtained from the active margin of a 3-day-old colony, was placed at the opposite end from the initial inoculation point of the plant pathogen. The plates were incubated for 10 days under controlled conditions to allow the antagonistic interaction between both organisms.

After the confrontation period, 10 5 mm diameter discs were taken along a straight line from the growth front of the plant pathogen toward the *Trichoderma* strip. The discs were transferred to Petri dishes containing fresh PDA and incubated for 2 weeks, with the growth of *Trichoderma* or the original plant pathogen recorded. The percentage of mycoparasitism (PP) was calculated using the equation proposed by Evans et al. (2003):

PP=(TG×100)/N

Where PP corresponds to the percentage of mycoparasitism, TG to the number of discs with *Trichoderma* growth, and N to the total number of discs evaluated per replication.

#### 2.6.3 Potential antagonism

The potential antagonism was estimated by integrating the results obtained from the mycoparasitism and antibiosis assays, recognizing that the biocontrol exerted by *Trichoderma* is a multifactorial process. Mycoparasitism (PP) evaluates the strain’s ability to establish direct contact, coil around, and lyse the pathogen’s hyphae, while antibiosis (PA) quantifies the inhibition of pathogen growth mediated by secondary metabolites (Santillan-Culquimboz et al., 2025).

To obtain a synthetic indicator of antagonistic performance, the average value of both mechanisms of action was calculated, according to the criterion proposed by Reyes-Figueroa et al. (2016).

Potential antagonism (AP) was calculated using the following expression:

AP=(PP+PA)/2

Where AP corresponds to potential antagonism, PP to the percentage of mycoparasitism, and PA to the percentage of antibiosis.

### 2.7 Data analysis

The antagonism assay data (antibiosis, mycoparasitism, and potential antagonism) were analyzed using a completely randomized design (CRD) with an analysis of variance (ANOVA), with five replications per treatment. Model assumptions were evaluated using residual analysis; normality was checked using the Shapiro–Wilk test and Q–Q plot inspection, while homogeneity of variances was assessed with Bartlett’s test. Multiple mean comparisons were performed using Tukey’s HSD test (*p* < 0.05). All statistical analyses and visualizations were conducted in the R environment (v. 4.5.2).

## 3 Results

### 3.1 Molecular identification

The pairwise similarity analysis of rpb2 and tef1-*α* sequences allowed the identification of 9 out of the 17 isolated strains, by comparing them with type sequences, according to the criteria proposed by Cai and Druzhinina (2021). These strains were assigned to the species *Trichoderma afroharzianum*, *T. parareesei*, *T. sexdentis*, *T. strigosum*, *T. reesei*, and *T. erinaceum*.

In the remaining strains, the nucleotide identity values did not reach the specific delimitation thresholds set (≥ 99% for *rpb2* and ≥ 97% for *tef1-α*), which prevented their assignment to previously described species (Table 2). In these cases, and in order to obtain a more robust taxonomic identification, a multilocus phylogenetic analysis was performed based on concatenated ITS, *rpb2*, and *tef1-α* sequences, allowing for the inference of their phylogenetic relationships and taxonomic position with greater resolution.

**Table 2:** Identification of *Trichoderma* strains through pairwise similarity analysis of rpb2 and tef1-α markers.

Isolate code	Reference species	rpb2 accession number	rpb2 identity (%)	tef1 accession number	tef1 identity (%)	Identification status
HWSC01	*T. sexdentis*	PQ334915	98.92%	PQ334916	96.91%	Not identified
HWSC02	*T. sexdentis*	PQ334915	98.72%	PQ334916	96.96%	Not identified
HWSC03	*T. sexdentis*	PQ334915	98.97%	PQ334916	97.49%	Not identified
HWSC04	*T. sexdentis*	PQ334915	98.91%	PQ334916	96.52%	Not identified
HWSC05	*T. sexdentis*	PQ334915	98.22%	PQ334916	96.33%	Not identified
HWSC06	*T. afroharzianum*	FJ442709	99.68%	FJ463301	99.25%	Identified
HWSC07	*T. afroharzianum*	FJ442709	99.78%	FJ463301	98.29%	Identified
HWSC10	*T. afarasin*	FJ442778	99.78%	FJ463400	88.41%	Not identified
HWSC11	*T. afarasin*	FJ442778	99.49%	FJ463400	84.95%	Not identified
HWSC12	*T. afarasin*	FJ442778	99.61%	FJ463400	88.43%	Not identified
HWSC13	*T. parareesei*	HM182963	99.78%	GQ354353	99.14%	Identified
HWSC16	*T. parareesei*	HM182963	99.76%	GQ354353	98.13%	Identified
HWSC18	*T. sexdentis*	PQ334915	99.91%	PQ334916	98.99%	Identified
HWSC19	*T. strigosum*	AF545556	100.00%	EU280019	99.62	Identified
HWSC21	*T. sexdentis*	PQ334915	99.91%	PQ334916	99.11%	Identified
HWSC26	*T. reesei*	HM182969	99.39%	KJ713193	99.64%	Identified
HWSC27	*T. erinaceum*	KJ842151	99.19%	AY750880	99.44%	Identified

### 3.2 Molecular phylogeny

**Figure 2:**
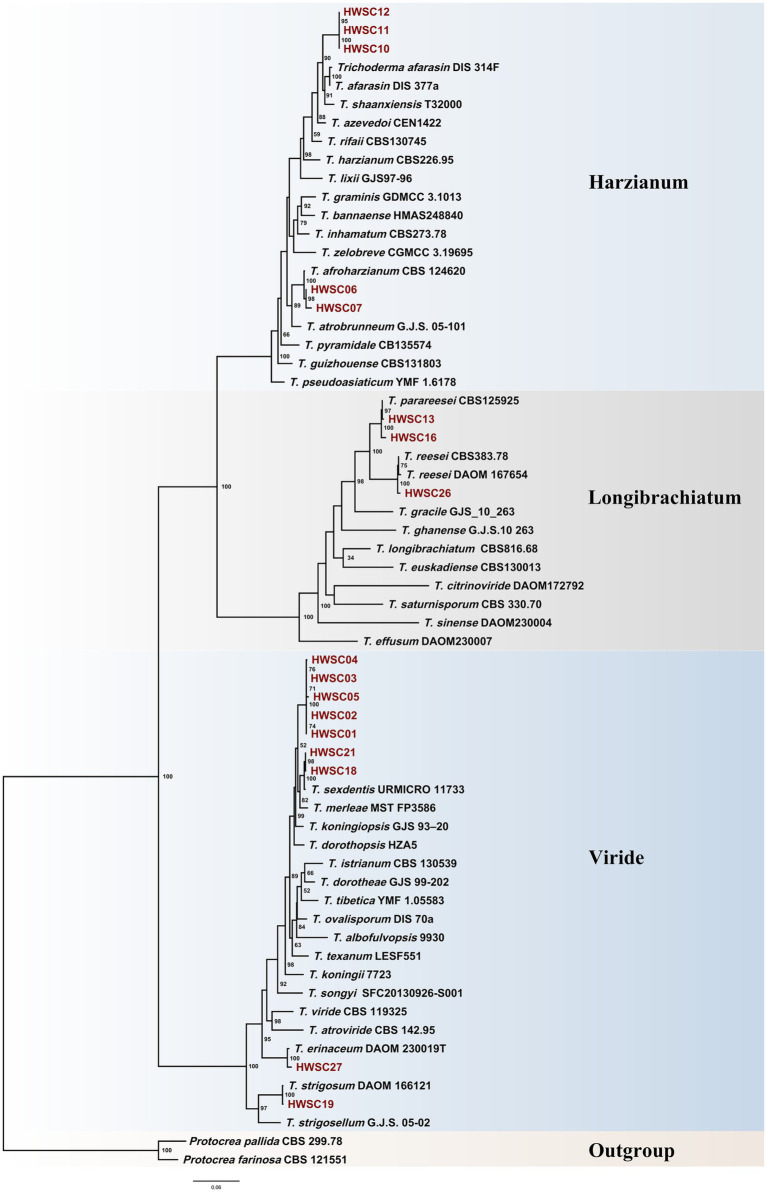
Phylogenetic tree inferred using the maximum likelihood (ML) method with concatenated ITS, *tef1-α*, and *rpb2* sequences. The strains isolated in the present study are highlighted in red.

The maximum likelihood (ML) phylogenetic analysis of the 17 isolates revealed their distribution in well-supported monophyletic clades, corresponding to the Harzianum, Longibrachiatum, and Viride. The topology obtained was congruent among the trees generated from the individual markers tef1-α and rpb2, as well as with the concatenated multilocus tree (ITS–tef1-α–rpb2), supporting the phylogenetic stability of the inferred groupings (Figure 2).

Several strains grouped with described species, including *Trichoderma afroharzianum* (HWSC06, HWSC07), *T. parareesei* (HWSC13, HWSC16), *T. reesei* (HWSC26), *T. sexdentis* (HWSC18, HWSC21), *T. strigosum* (HWSC19), and *T. erinaceum* (HWSC27), all with high bootstrap support.

In contrast, the strains HWSC01, HWSC02, HWSC03, HWSC04, and HWSC05 formed a monophyletic subclade sister to *T. sexdentis*, with moderate to high support, but clearly differentiated from this species. Since their phylogenetic affiliation could not be resolved conclusively and the multilocus identity values did not reach the specific delimitation thresholds, these isolates are provisionally reported as *Trichoderma* cf. *sexdentis*, following the taxonomic criteria proposed by Cai and Druzhinina (2021).

Additionally, the isolates HWSC10, HWSC11, and HWSC12 were positioned in a phylogenetically differentiated group within the Harzianum clade, with no close association to described species, suggesting that they may correspond to a potential new species. Therefore, for this study, they are reported as *Trichoderma* sp. However, their formal delimitation will require additional morphological, genomic, and ecological evidence.

### 3.3 Morphological characterization

*Trichoderma afroharzianum* P. Chaverri, F. B. Rocha, Degenkolb and I. Druzhinina, Mycologia 107: 568 (2015).

On PDA, the colonies developed abundant, cottony, white aerial mycelium during the initial growth phases; later, the margin became diffuse and denser, with grayish tones toward the periphery and brown in the center. From the sixth day, peripheral sporulation was observed, forming a layer of conidiophores with a dusty appearance. On SNA, the growth was thinner and radial, with evident sporulation showing a dark green central color and progressively lighter edges.

**Figure 3:**
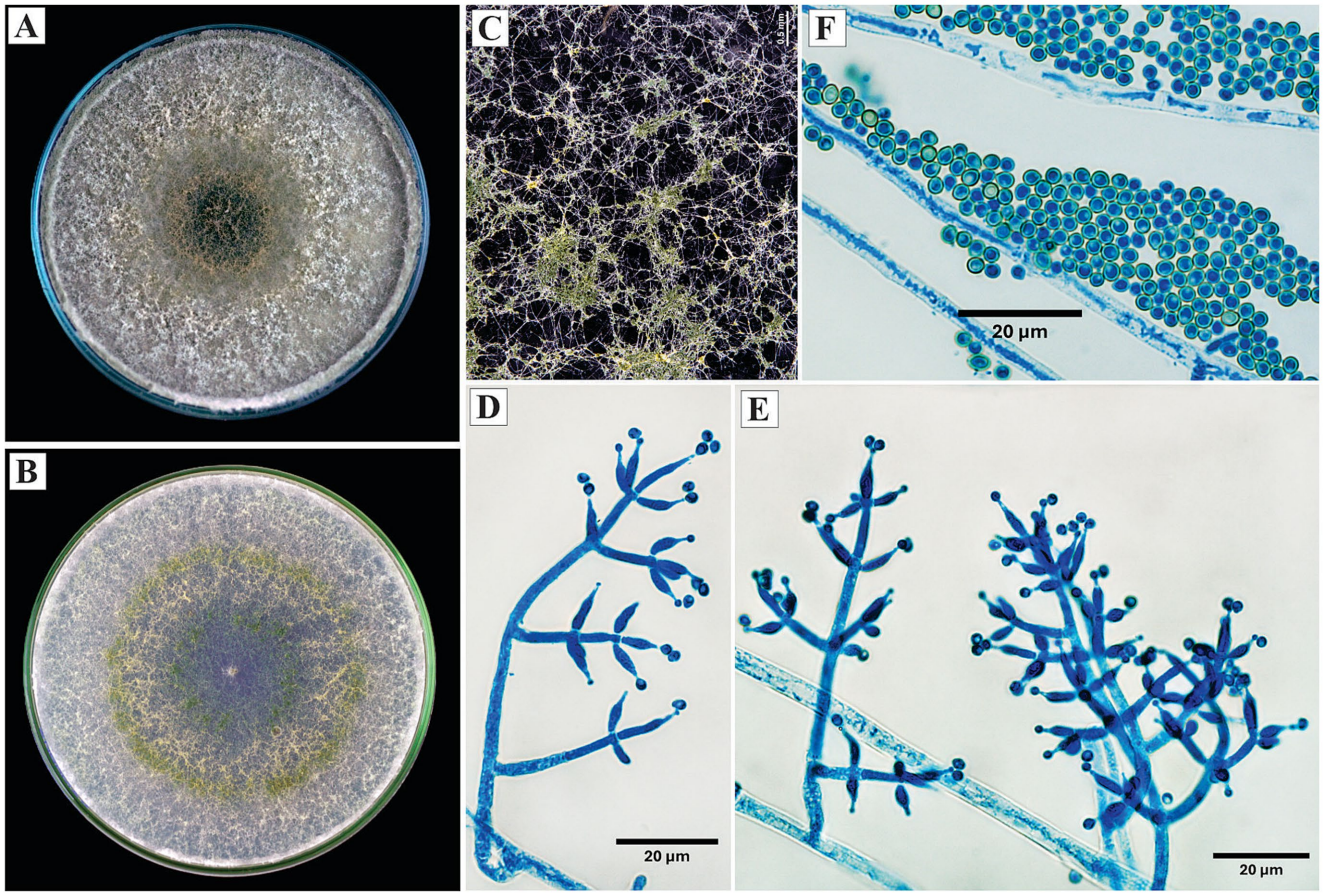
Macroscopic and microscopic morphology of *Trichoderma afroharzianum*. **(A)** Colony on PDA. **(B)** Colony on SNA. **(C)** Pustules on SNA medium. **(D,E)** Conidiophores. **(F)** Conidia. The macroscopic photos **(A–C)** correspond to 10 days of incubation.

Microscopically, the conidiophores exhibited opposite branching, with verticils of 2 to 5 phialides. The phialides were lageniform to ampulliform, measuring 7.8 to 11.9 μm in length. The conidia were subglobose to ovoid, with an average size of 2.6 × 2.8 μm (Figure 3).

*Specimens examined*: Peru, Amazonas region, Utcubamba, 5°44’49.8”S, 78°21’41.0”W, isolated from cacao xylem, 2024, H. W. Santillan-Culquimboz. Code: HWSC06, HWSC07.

*Trichoderma erinaceum* Bissett, Kubicek and Szakacs, Canadian Journal of Botany 81: 584 (2003).

On PDA, the colonies exhibited dense, cottony, white mycelium with radial growth and no visible sporulation. On SNA, the mycelium was thinner, with evident sporulation toward the periphery, manifested as yellowish granular pustules.

**Figure 4:**
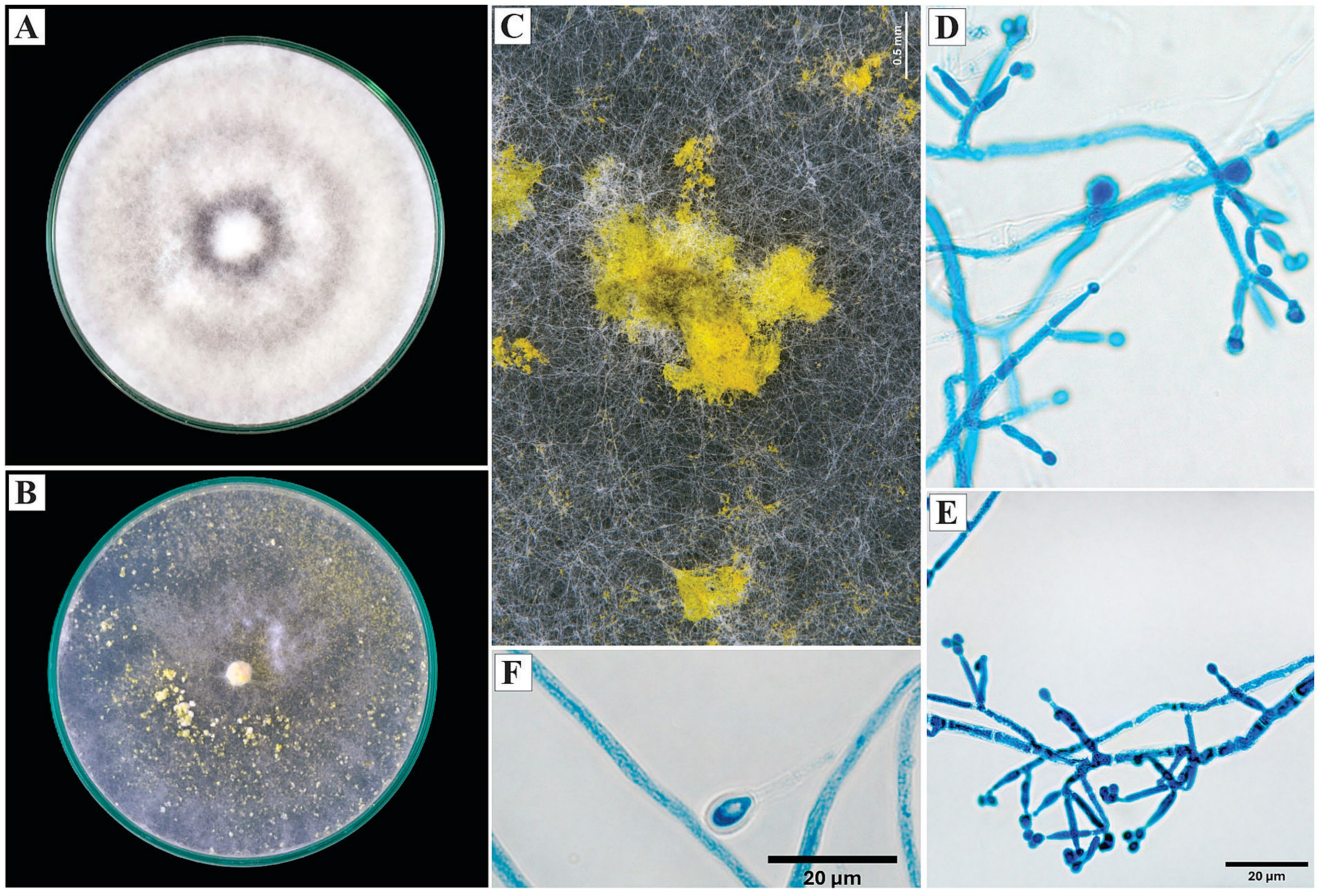
Macroscopic and microscopic morphology of *Trichoderma erinaceum*. **(A)** Colony on PDA. **(B)** Colony on SNA. **(C)** Pustules on SNA medium. **(D,E)** Conidiophores. **(F)** Chlamydospore. The macroscopic photos **(A–C)** correspond to 10 days of incubation.

Microscopically, the conidiophores were straight and erect, with verticils of 2 to 3 lageniform phialides up to 12 μm in length. The conidia were subglobose to ovoid, with an average diameter of 4 μm. Subglobose chlamydospores were observed (Figure 4).

*Specimen examined*: Peru, Amazonas region, Condorcanqui, 4°33’27”S, 77°52’22”W, isolated from cacao xylem, 2024, H. W. Santillan-Culquimboz. Code: HWSC27.

*Trichoderma parareesei* Atanasova et al., Applied and Environmental Microbiology 76: 6710–6,721 (2010).

On PDA, the colonies developed abundant, cottony, yellowish aerial mycelium; sporulation was moderate from the sixth day, with dispersed green granules, denser toward the center, accompanied by yellow pigmentation of the medium. On SNA, the growth was radial and less dense, with sporulation concentrated at the periphery, forming green pustules with white edges.

**Figure 5:**
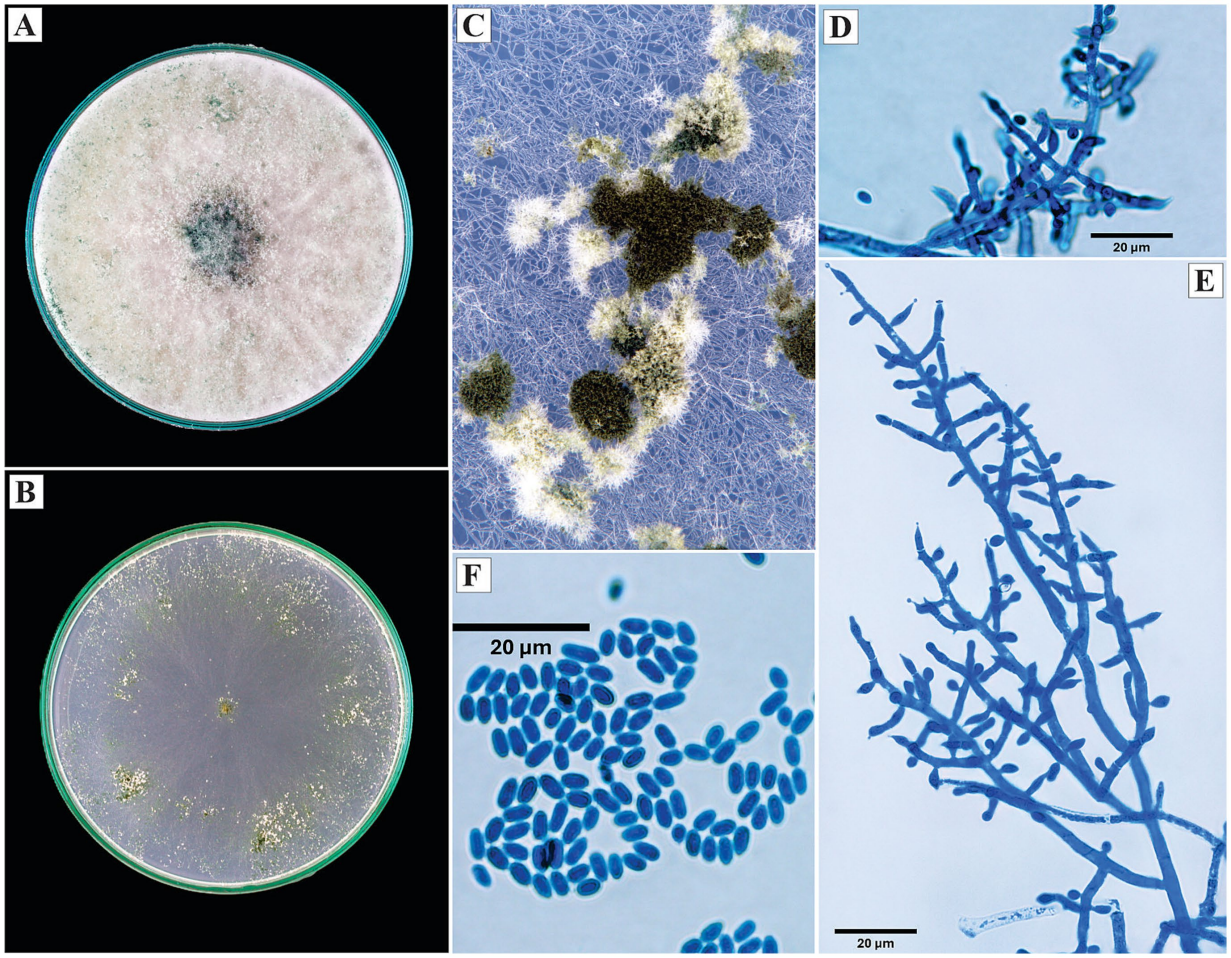
Macroscopic and microscopic morphology of *Trichoderma parareesei*. **(A)** Colony on PDA. **(B)** Colony on SNA. **(C)** Pustules formed on SNA medium. **(D,E)** Conidiophores observed under the microscope. **(F)** Detail of the conidia. The macroscopic photos **(A–C)** correspond to 10 days of incubation.

The conidiophores were narrow, with verticils of 2 to 3 intercalary phialides. The phialides were lageniform to ampulliform, measuring 8 to 10 μm in length. The conidia were ellipsoidal, with an average size of 2.5 × 4.9 μm (Figure 5).

*Specimens examined*: Peru, Amazonas region, Bagua, isolated from cacao xylem and roots, 2024, H. W. Santillan-Culquimboz. Code: HWSC13 (5°43’20”S, 78°25’00”W), HWSC16 (5°09’37”S, 78°20’41”W).

*Trichoderma reesei* E. G. Simmons, 2nd International Mycological Congress (Tampa): 618 (1977).

On PDA, the colonies developed dense, cottony, and radial growth mycelium, with no visible sporulation after 10 days of incubation and a slight yellow pigmentation of the medium. On SNA, the growth was thinner and less dense, with no formation of pustules or evident sporulation during the observation period.

**Figure 6:**
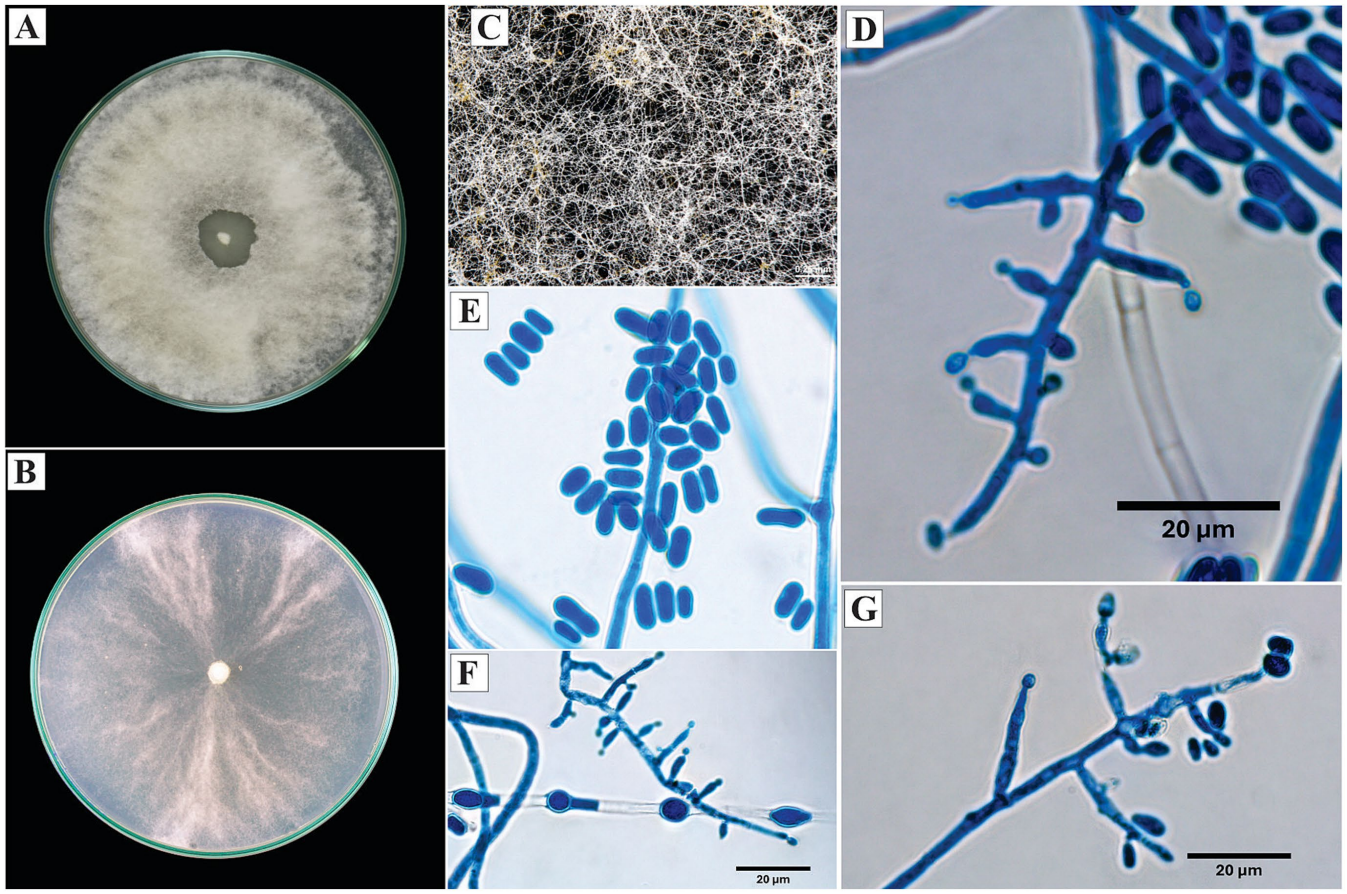
Macroscopic and microscopic morphology of *Trichoderma reesei*. **(A)** Colony on PDA. **(B)** Colony on SNA. **(C)** Mycelium on SNA medium. **(D,F,G)** Conidiophores observed under the microscope. **(E)** Conidia. The macroscopic photos **(A–C)** correspond to 10 days of incubation.

Microscopically, the conidiophores exhibited sparse branching and mostly intercalary phialides. The phialides were lageniform, generally straight, with lengths ranging from 8 to 10 μm. The conidia were ellipsoidal, with variable sizes ranging from 4.0 to 7.9 μm in length and 2.7 to 4.0 μm in width(Figure 6).

*Specimen examined*: Peru, Amazonas region, Condorcanqui, 4°32′23″S, 77°53′54″W, isolated from cacao xylem, 2024, H. W. Santillan-Culquimboz. Code: HWSC26.

*Trichoderma sexdentis* L. S. Sales, J. T. de Souza & P. A. S. Marbach, Persoonia 54: 572 (2025).

On PDA, the colonies developed cottony white mycelium during the initial phases; from the sixth day, they acquired a light green color associated with sporulation, with no evident diffusible pigmentation. On SNA, the mycelium was less dense, with dense sporulation starting on the tenth day, predominantly green, and the pustules were dense and flat.

**Figure 7:**
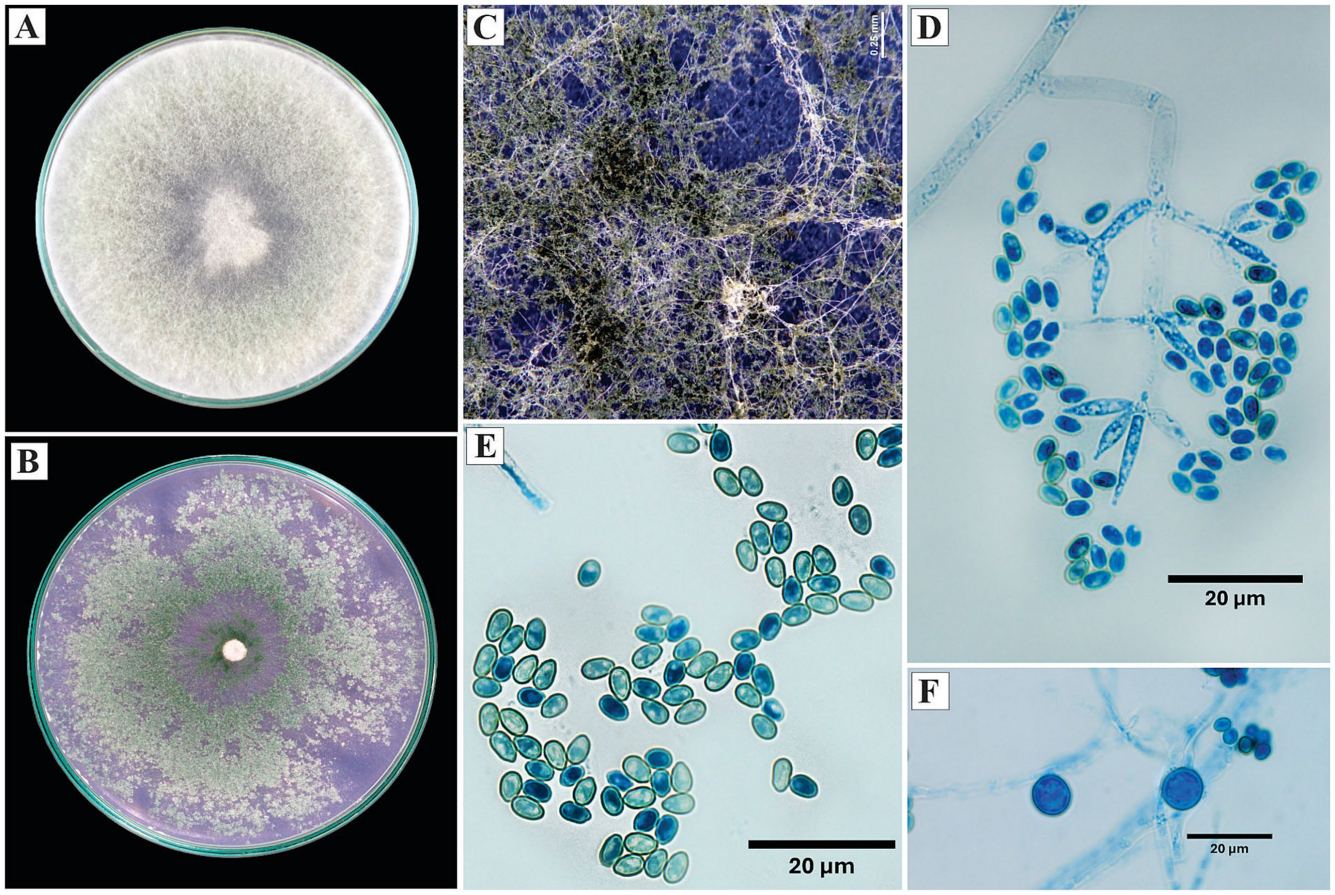
Macroscopic and microscopic morphology of *Trichoderma sexdentis*. **(A)** Colony on PDA. **(B)** Colony on SNA. **(C)** Pustules formed on SNA medium. **(D,E)** Conidia observed under the microscope. **(F)** Chlamydospore. The macroscopic photos **(A–C)** correspond to 10 days of incubation.

Microscopically, the conidiophores were branched, with a subpyramidal architecture, and the conidia were arranged in clusters toward the distal portion. Verticils of 2 to 4 phialides were observed; the phialides were ampulliform and opposite, with lengths of 9.6 μm in lateral phialides and an average length of 14.7 μm in terminal phialides. The conidia were ellipsoidal to ovoid, measuring 2.6 × 4.7 μm. Globose chlamydospores were observed (Figure 7).

*Specimens examined*: Peru, Amazonas region, Condorcanqui, 4°35′38″S, 77°51′17″W, isolated from cacao xylem and branches, 2024, H. W. Santillan-Culquimboz. Codes: HWSC18, HWSC21.

*Trichoderma strigosum* Bissett, Canadian Journal of Botany 69: 2373–2,417 (1991).

On PDA, the colony developed cottony aerial mycelium with radial growth; from the sixth day, the formation of a central green ring was observed, indicative of sporulation. On SNA, the mycelium was less dense, with sporulation manifested as solitary green pustules mainly distributed toward the periphery of the plate.

**Figure 8:**
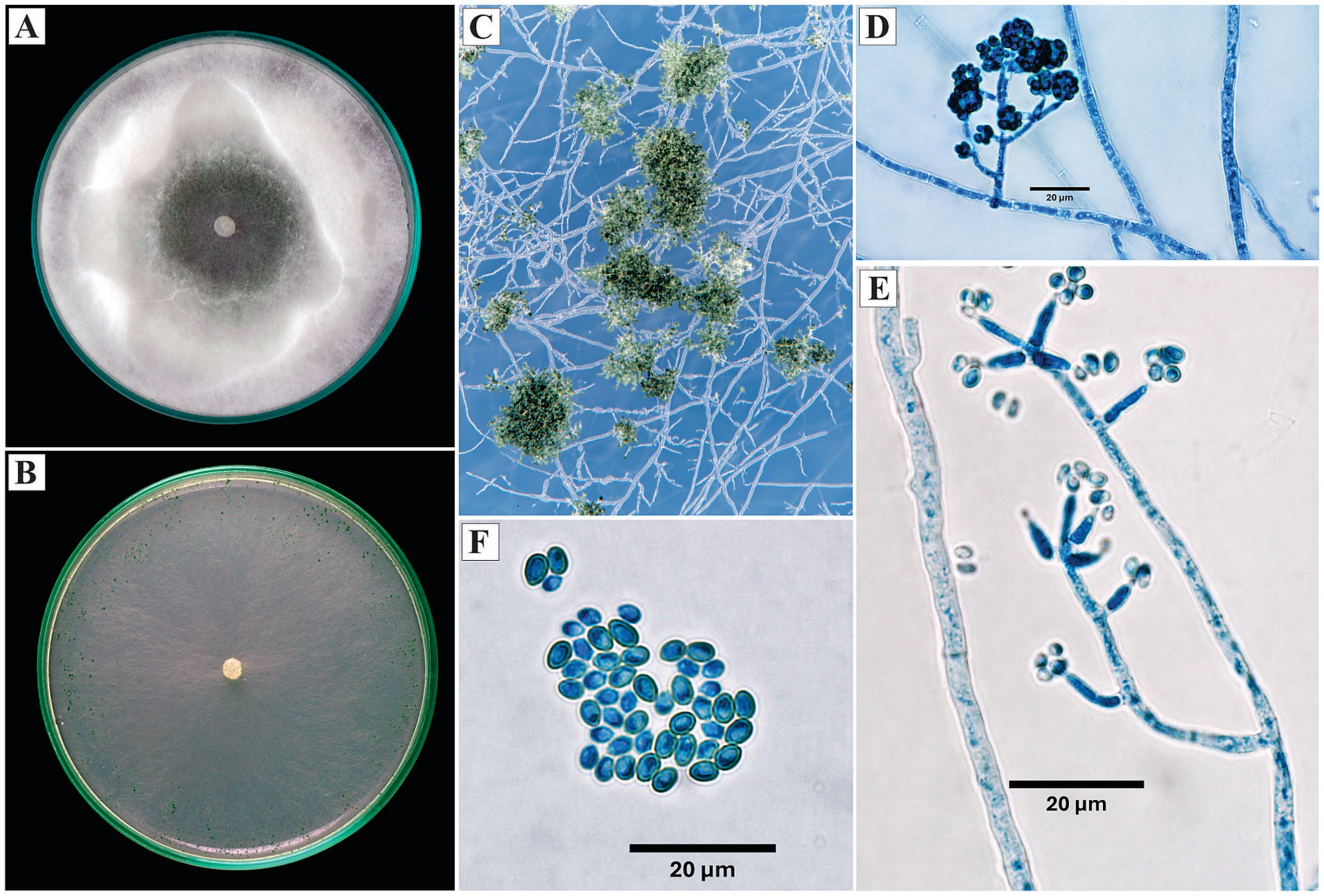
Macroscopic and microscopic morphology of *Trichoderma strigosum*. **(A)** Colony on PDA. **(B)** Colony on SNA. **(C)** Pustules on SNA medium. **(D)** Conidiophores with conidia. **(E)** Conidiophores. **(F)** Conidia. The macroscopic photos **(A–C)** correspond to 10 days of incubation.

The conidiophores were branched, with conidia arranged in compact groups on the branches of the conidiophore. The phialides were ampulliform to lageniform, opposite, up to 12 μm in length. The conidia were ellipsoidal, smooth, with an average length of 4.3 μm (Figure 8).

*Specimen examined*: Peru, Amazonas region, Condorcanqui, 4°35’38”S, 77°51’17”W, isolated from cacao xylem, 2024, H. W. Santillan-Culquimboz. Code: HWSC19.

#### 3.3.1 *Trichoderma* cf. sexdentis

On PDA, the colonies exhibited dense, cottony, radial mycelium, with no visible sporulation after 10 days. On SNA, the mycelium was less dense, with green sporulation distributed across the plate and concentrated toward the periphery, where granular pustules formed.

**Figure 9:**
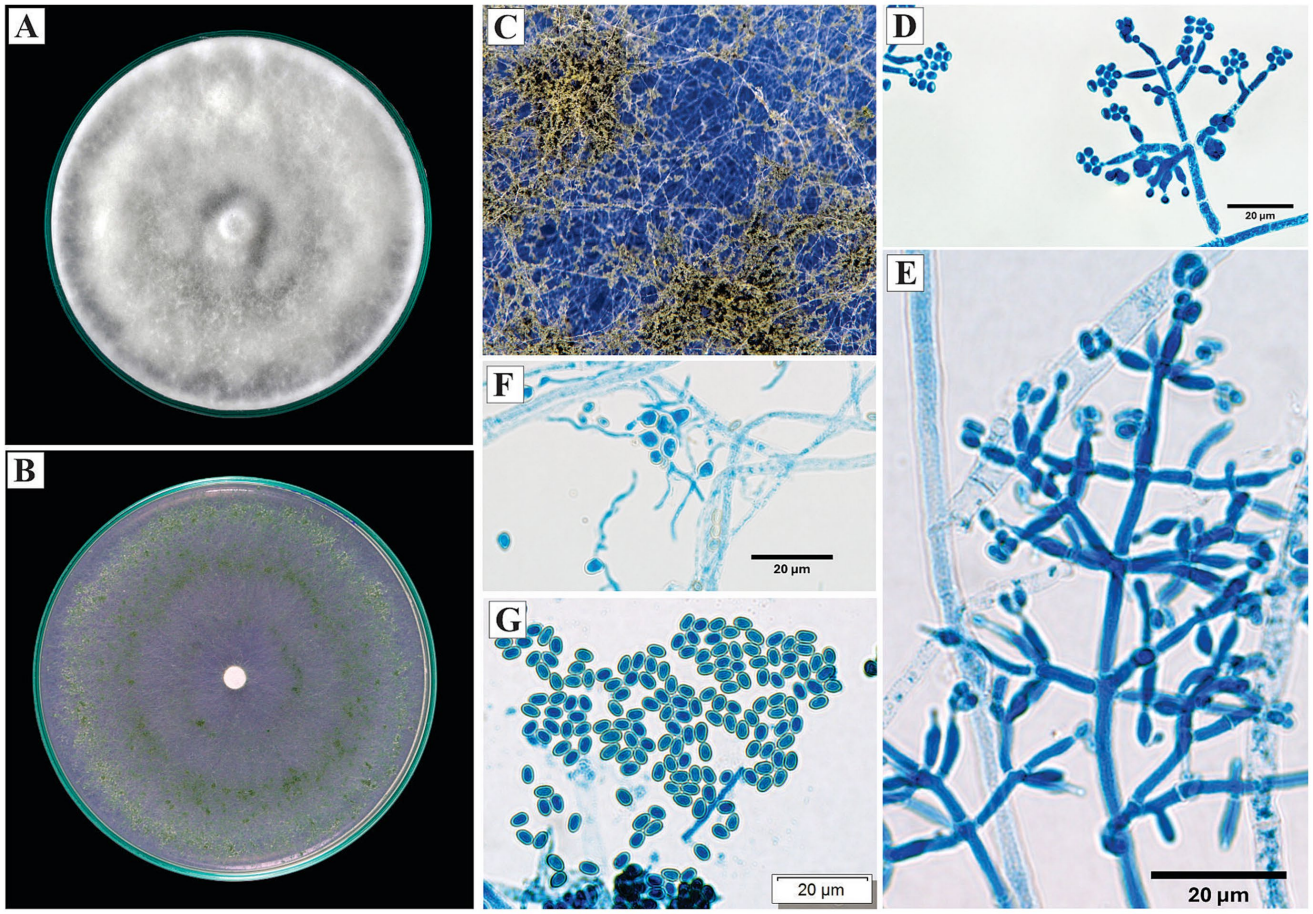
Macroscopic and microscopic morphology of *Trichoderma* cf*. sexdentis*. **(A)** Colony on PDA. **(B)** Colony on SNA. **(C)** Pustules formed on SNA medium. **(D,E)** Conidiophores observed under the microscope. **(F)** Chlamydospores. **(G)** Conidia. The macroscopic photos **(A–C)** correspond to 10 days of incubation.

The conidiophores were erect and branched, with a well-defined main axis and subpyramidal architecture; the branching was irregular to verticillate, with verticils of 2–4 phialides arranged at nearly right angles. The phialides were lageniform to ampulliform, slightly curved, with terminal phialides up to 16.5 μm and lateral ones approximately 12 μm. The conidia were ellipsoidal to ovoid, smooth and hyaline, measuring 4.1 × 2.5 μm. Globose to subglobose chlamydospores were observed (Figure 9).

The provisional assignment is based on morphological and phylogenetic affinities with *T. sexdentis*, although consistent divergences in the dimensions of the phialides were detected.

*Specimens examined*: Peru, Amazonas region, Utcubamba, 5°44’10”S, 78°24’45”W, isolated from cacao xylem, 2024, H. W. Santillan-Culquimboz. Codes: HWSC01, HWSC02, HWSC03, HWSC04, HWSC05 (5°44’49.8”S, 78°21’41.0”W).

#### 3.3.2 *Trichoderma* sp.

On PDA, the colonies developed dense, cottony, and radial aerial mycelium; sporulation was observed from the tenth day as white to grayish pustules initially in the center, which spread toward the periphery. On SNA, the mycelium was thinner, with sporulation concentrated in the central region and a green to light green color.

**Figure 10:**
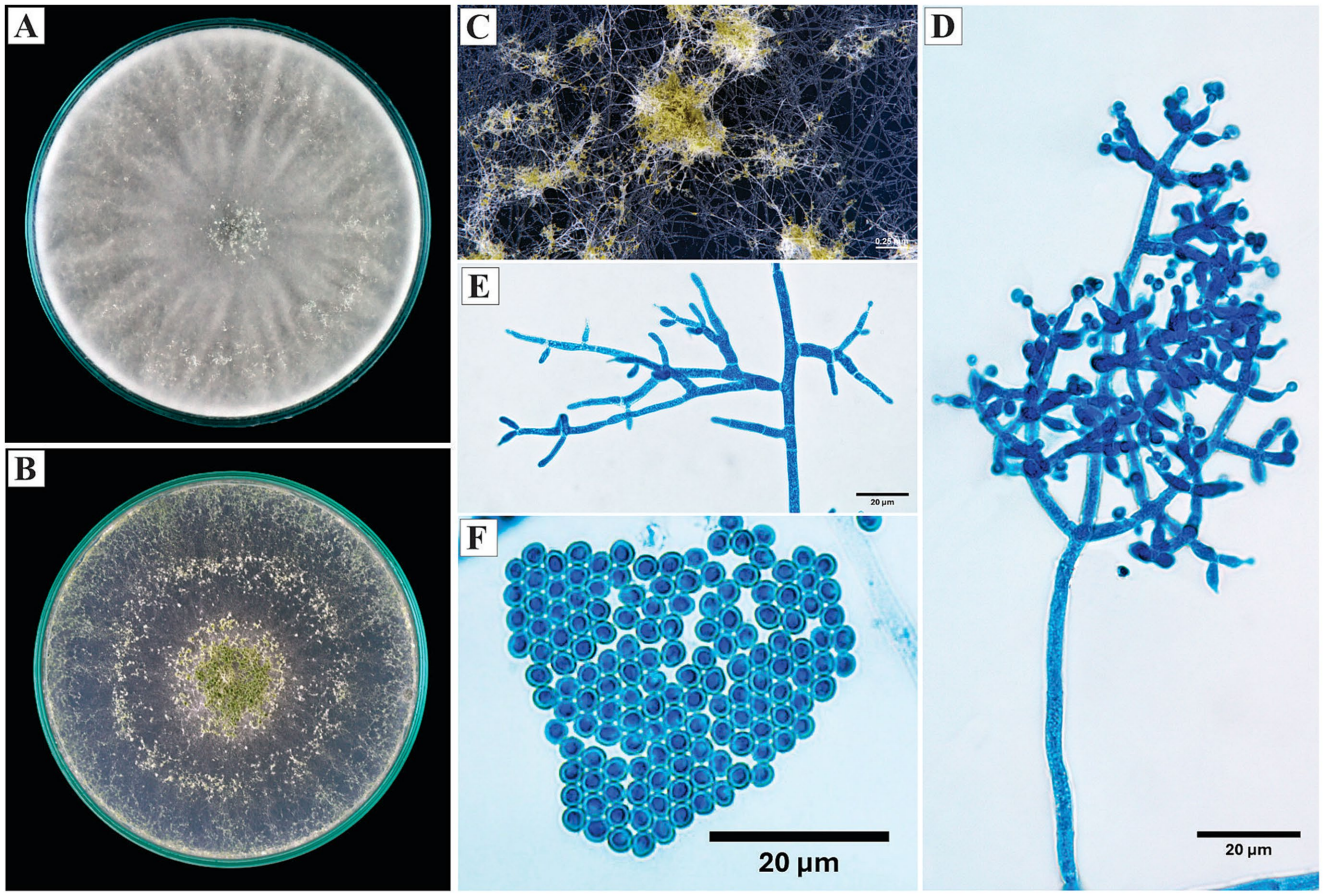
Macroscopic and microscopic morphology of *Trichoderma* sp. **(A)** Colony on PDA. **(B)** Colony on SNA. **(C)** Pustules observed on SNA medium. **(D)** Conidiophore observed under the microscope. **(E)** Conidiophore formation. **(F)** Conidia. The macroscopic photos **(A–C)** correspond to 10 days of incubation.

The conidiophores were loose, irregularly branched, with reduced verticils of 2 to 4 phialides. The phialides were lageniform to ampulliform, with an average length of 9 μm. The conidia were globose to subglobose, smooth and hyaline, with an average diameter of 2.7 μm (Figure 10).

*Specimens examined*: Peru, Amazonas region, Bagua, 5°43’20”S, 78°25’00”W, isolated from cacao xylem, 2024, H. W. Santillan-Culquimboz. Codes: HWSC10, HWSC11, HWSC12.

### 3.4 Antagonist activity

Endophytic isolates of *Trichoderma* exhibited antagonistic activity against *Fusarium* sp. and *Moniliophthora roreri*, associated with the expression of antibiosis and mycoparasitism mechanisms. These mechanisms were evidenced through the inhibition of mycelial growth and direct colonization of the pathogen’s mycelium. However, the expression and magnitude of these mechanisms was not homogeneous, but rather strongly depended on the specific interaction between each strain and the evaluated plant pathogen. This variability was reflected in contrasting patterns of antagonistic efficacy, both between species and among phylogenetically related strains, highlighting differences in antagonistic performance between species and strains of the *Trichoderma* genus.

#### 3.4.1 Antibiosis

Against *Fusarium* sp., the inhibition percentages by antibiosis ranged from 17.78 to 39.61%, while against *M. roreri*, globally higher values were recorded, ranging from 29.68 to 60.99%. The strains HWSC01 and HWSC03, assigned to *Trichoderma cf. sexdentis*, showed high and consistent inhibitory activity against both plant pathogens, with values close to 40% against *Fusarium* sp. and exceeding 55% against *M. roreri*, demonstrating a relatively stable antibiotic profile.

In contrast, *T. erinaceum* (HWSC27) showed a response strongly dependent on the plant pathogen, with moderate inhibition against *Fusarium* sp. (≈32%) and the highest level of antibiosis recorded against *M. roreri* (≈61%). An inverse pattern was observed in *Trichoderma* sp. (HWSC11), whose inhibition against *Fusarium* sp. was high (≈38%), comparable to that of the most active strains of *T. cf. sexdentis*, while against *M. roreri* it reached intermediate values (≈39%).

**Figure 11:**
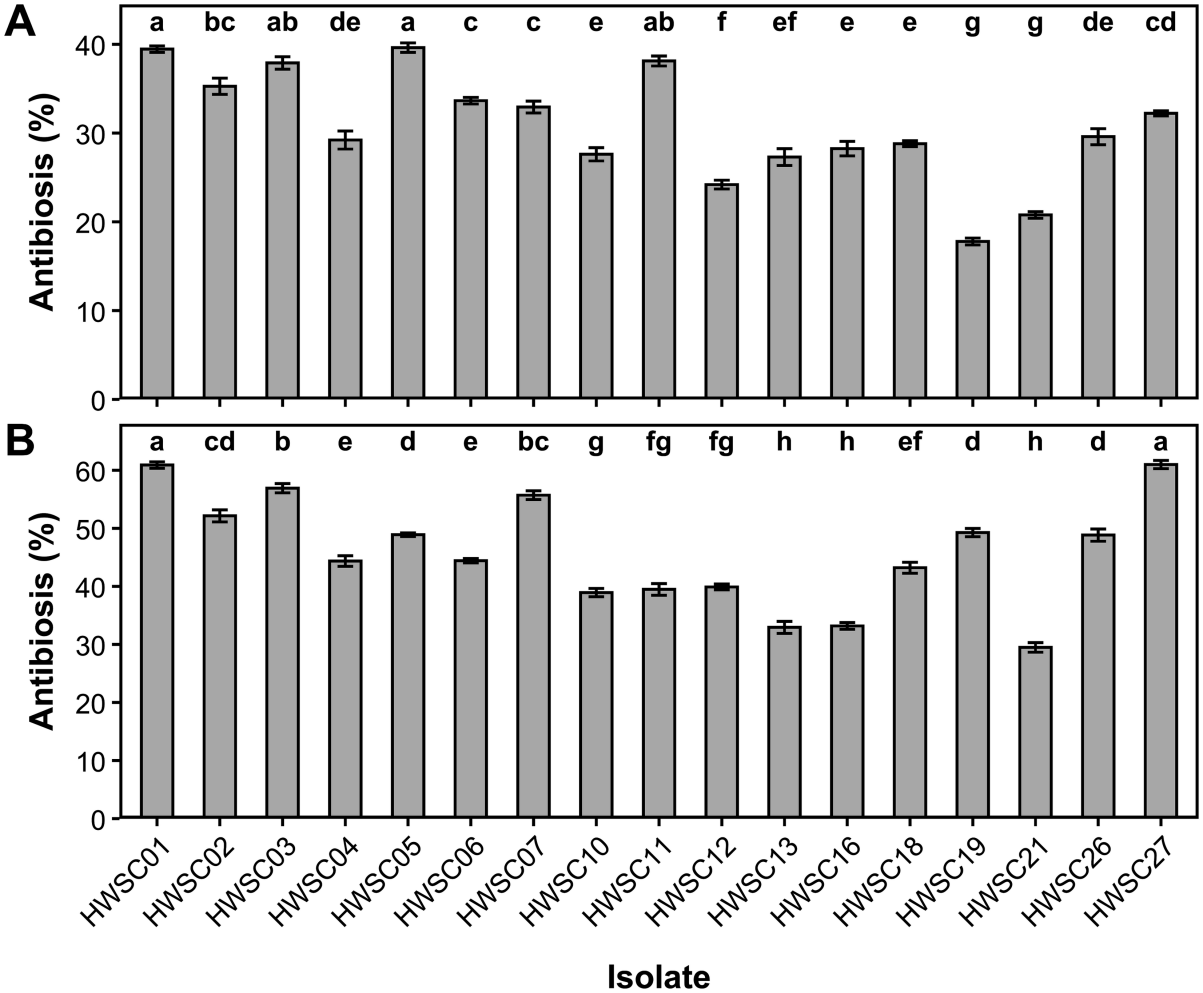
Antibiosis exerted by endophytic *Trichoderma* strains. **(A)** Antibiosis against *Fusarium* sp. **(B)** Antibiosis against *Moniliophthora roreri*. Different letters indicate statistically significant differences among isolates (Tukey test, *p* < 0.05).

Similarly, *T. strigosum* (HWSC19) exhibited a contrasting behavior, with moderate inhibition against *M. roreri* (≈50%) and the lowest value against *Fusarium* sp. (≈18%). In *T. sexdentis*, the strain HWSC18 showed intermediate levels of inhibition against both plant pathogens, while HWSC21 consistently ranked among the strains with the lowest antibiotic activity. Meanwhile, *T. parareesei* (HWSC13 and HWSC16) recorded the lowest inhibition values against both pathogens, while the remaining strains were distributed within intermediate ranges (Figure 11).

#### 3.4.2 Mycoparasitism

Several strains proved to be aggressive mycoparasites of the evaluated plant pathogens. The isolates HWSC01, HWSC02, HWSC03, and HWSC05, corresponding to *Trichoderma cf. sexdentis*, along with *Trichoderma* sp. (HWSC11) and *T. sexdentis* (HWSC18), achieved colonization levels above 90% against both plant pathogens, reflecting high and consistent mycoparasitic behavior.

Other strains showed a pathogen-dependent response. *T. strigosum* (HWSC19) exhibited high colonization against *Fusarium* sp. (96%), but intermediate performance against *M. roreri* (74%). Similarly, *T. erinaceum* (HWSC27) achieved full colonization against *M. roreri* (100%), while its mycoparasitic capacity against *Fusarium* sp. was moderate (64%).

**Figure 12:**
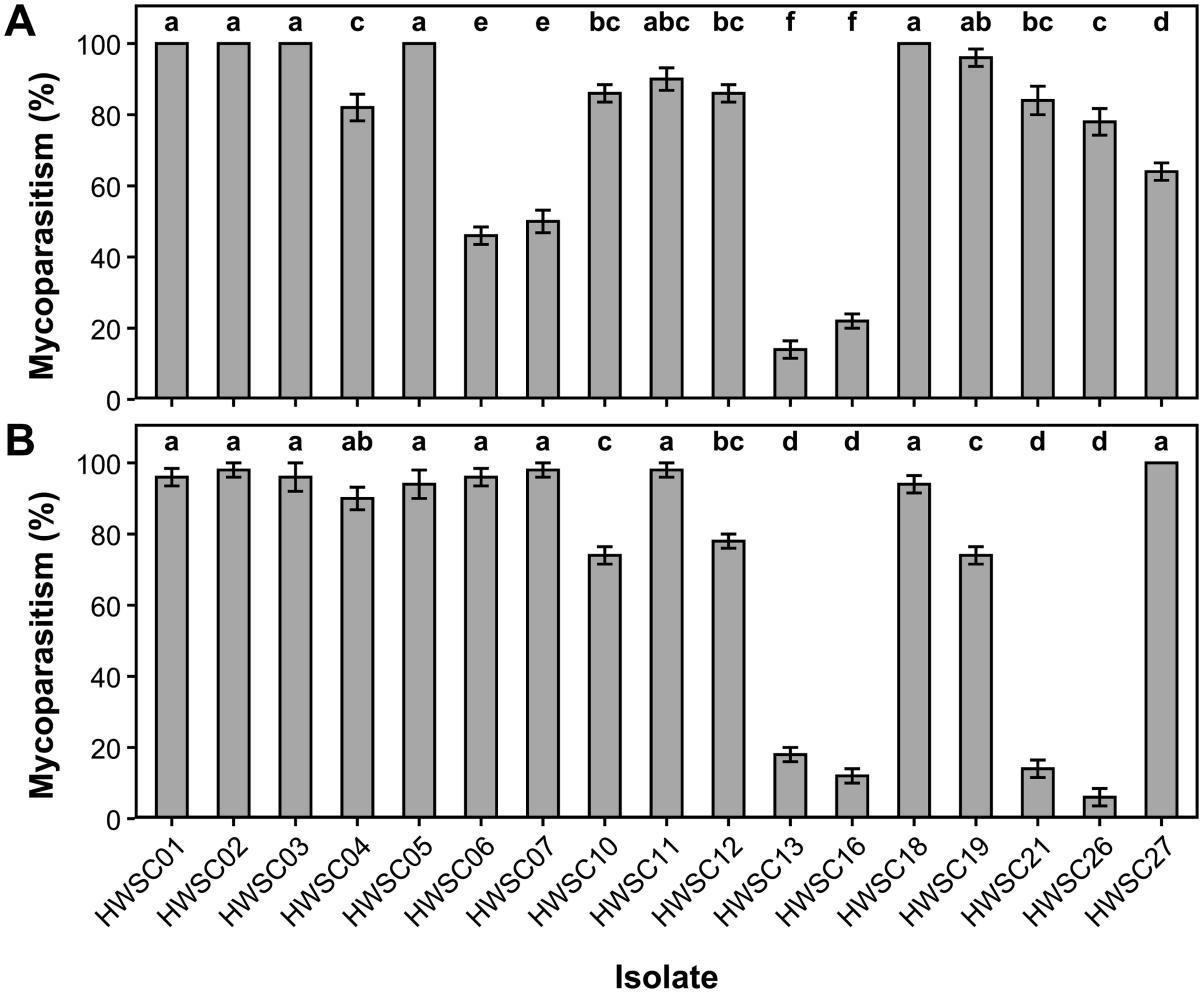
Mycoparasitism exerted by endophytic *Trichoderma* strains. **(A)** Mycoparasitism against *Fusarium* sp. **(B)** Mycoparasitism against *Moniliophthora roreri*. Different letters indicate statistically significant differences among isolates (Tukey test, *p* < 0.05).

A comparable pattern was observed in *T. afroharzianum* (HWSC06 and HWSC07), whose strains ranked among the most active against *M. roreri*, with mycoparasitism levels of 96 and 98%, respectively, but showed intermediate values against *Fusarium* sp. (46–50%). In contrast, *T. sexdentis* (HWSC21) and *T. reesei* (HWSC26) exhibited greater colonization against *Fusarium* sp. (84 and 78%, respectively) than against *M. roreri*, where values remained below 15%. *T. parareesei* (HWSC13 and HWSC16) recorded the lowest mycoparasitism levels against both plant pathogens, while the remaining strains were distributed within intermediate ranges (Figure 12).

#### 3.4.3 Potential antagonism

The potential antagonism of the endophytic *Trichoderma* strains showed contrasting patterns and a marked dependence on the evaluated plant pathogen. The strains HWSC01, HWSC02, HWSC03, and HWSC05, assigned to *Trichoderma cf. sexdentis*, maintained moderate to high potential antagonism values, exceeding 67% against *Fusarium* sp. and over 71% against *M. roreri*.

A similar behavior was observed in *Trichoderma* sp. (HWSC11) and *T. sexdentis* (HWSC18), which reached intermediate values exceeding 64% against both plant pathogens. In contrast, *T. erinaceum* (HWSC27) showed a pathogen-dependent response, with intermediate antagonism against *Fusarium* sp. (≈50%) and the highest values recorded against *M. roreri*, close to 80%. In agreement, *T. afroharzianum* (HWSC06 and HWSC07) showed low values against *Fusarium* sp., but a notable increase against *M. roreri* (70–76%).

**Figure 13:**
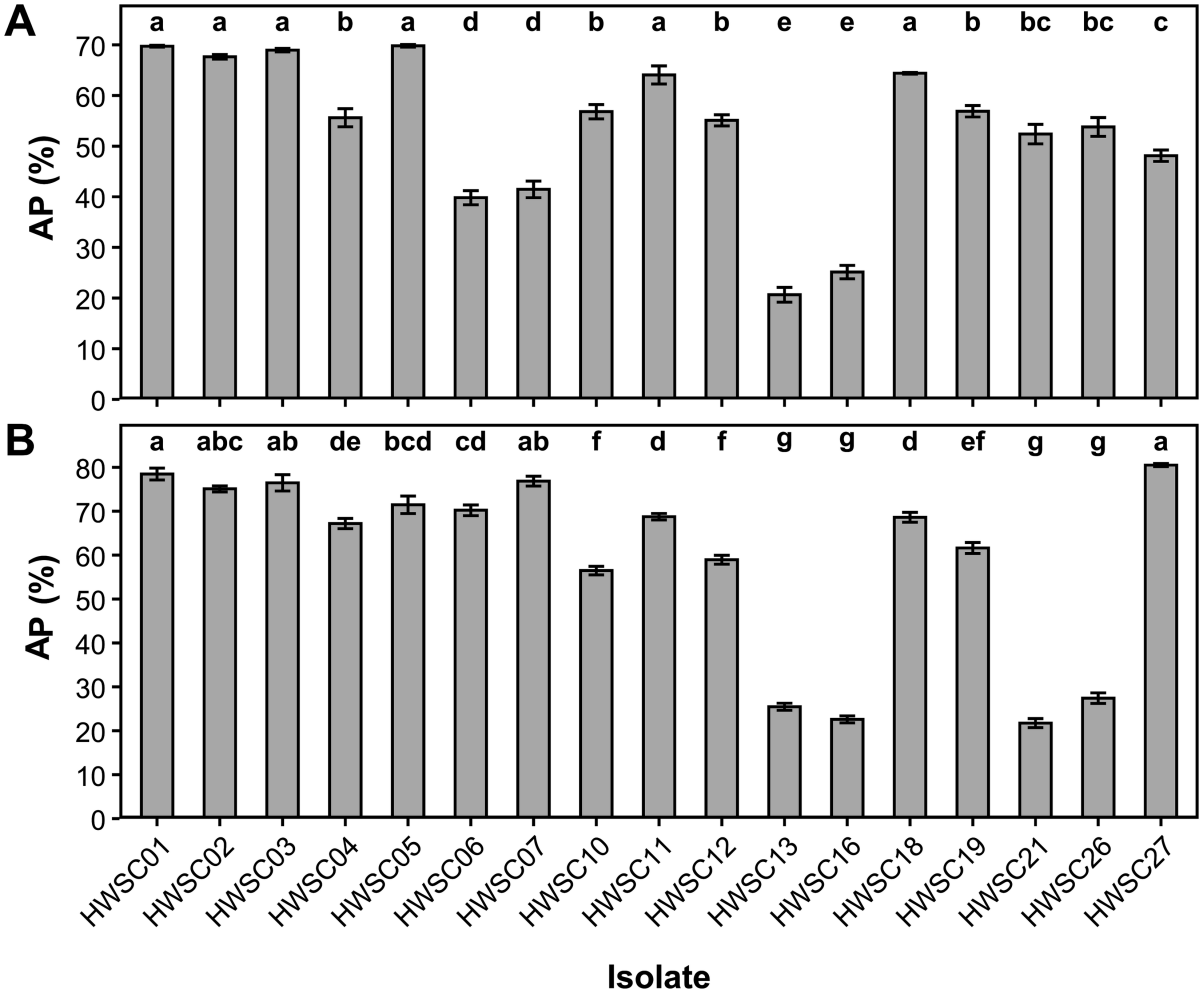
Antagonistic potential (AP) of endophytic *Trichoderma* strains. **(A)** Antagonism against *Fusarium* sp. **(B)** Antagonism against *Moniliophthora roreri*. Different letters indicate statistically significant differences among isolates (Tukey test, *p* < 0.05).

In turn, *T. sexdentis* (HWSC21) recorded moderate values against *Fusarium* sp. (≈52%) and the lowest level against *M. roreri* (≈22%). *T. parareesei* (HWSC13 and HWSC16) remained among the strains with the lowest potential antagonism, with values close to 20% against both plant pathogens, while the other strains were distributed within intermediate ranges (Figure 13).

## 4 Discussion

Since its first taxonomic description by Persoon in 1794, the genus *Trichoderma* has been the subject of extensive research, driven by its high species diversity and functional importance in agricultural and biotechnological contexts (Mukhopadhyay and Kumar, 2020). In particular, accurate taxonomic delimitation of its species is an essential requirement for the selection, validation, and safe application of biocontrol agents (Jambhulkar et al., 2024). However, identification based solely on morphological traits has proven insufficient in this genus, due to the low phenotypic variation and structural simplicity that characterize many cryptic species (Bustamante et al., 2021). Therefore, molecular approaches have taken a central role in *Trichoderma* taxonomy (Nascimento Brito et al., 2023).

In this study, endophytic *Trichoderma* strains were identified through molecular pairwise comparison and multilocus analysis (ITS, *tef1-α*, and *rpb2*), complemented by morphological characterization. The markers *tef1-α* and *rpb2* proved effective in providing sufficient interspecific variability and defined similarity thresholds for the accurate delimitation of some of the isolates (Cai and Druzhinina, 2021). However, in the remaining cases, it was not possible to achieve accurate species-level identification. Although multilocus phylogenetic analysis allowed for the inference of the evolutionary relationships of these isolates and their affinity with certain clades, the lack of sufficient resolution and the failure to meet the established similarity thresholds prevented their definitive assignment to previously described species, suggesting the presence of species still uncharacterized within the *Trichoderma* genus (Figure 13).

The strains assigned as *Trichoderma cf. sexdentis* formed a monophyletic subclade sister to *T. sexdentis* within the Koningii clade, a species recently described by Crous et al. (2025). This close phylogenetic affinity, in the absence of sufficient evidence for a definitive taxonomic assignment, could be explained by incomplete lineage sorting and recombination processes, phenomena common in fungi with recent diversification; however, gene duplication and loss, horizontal gene transfer, and hybridization could also explain this phenomenon (Bustamante et al., 2021). Additionally, the presence of slight morphological discrepancies, inconclusive by themselves, is consistent with the close phylogenetic affinity and supports a conservative interpretation of these isolates as *T. cf. sexdentis*.

Additionally, the strains HWSC10, HWSC10, and HWSC12 formed an independent lineage within the Harzianum clade, with no clear affinity to described species, suggesting the possible presence of an uncharacterized species. This pattern is consistent with the high complexity of the Harzianum clade, one of the most diverse in the genus, where morphological heterogeneity and the phylogenetic proximity between species hinder precise taxonomic delimitation (Cai and Druzhinina, 2021; Cao et al., 2022; Zhao et al., 2023). However, these results will need to be complemented with additional analyses to more accurately assess the taxonomic status of this lineage.

On the other hand, the identified species showed morphological consistency with the descriptions previously reported in the literature, supporting the robustness of the taxonomic identification performed. In particular, the characteristics observed in *Trichoderma afroharzianum*, *T. parareesei*, *T. sexdentis*, *T. strigosum*, *T. reesei*, and *T. erinaceum* matched the diagnostic traits described by previous studies (Bissett, 1991; Atanasova et al., 2010; Jambhulkar et al., 2024; Crous et al., 2025), reinforcing the consistency between the morphological and molecular evidence in these species.

Given that studies on endophytic *Trichoderma* associated with cacao in Peru are still limited, this work constitutes the first report of *Trichoderma sexdentis* and *T. strigosum* as endophytes of *Theobroma cacao* in this geographic context. The detection of these taxa, along with the presence of phylogenetically unresolved species, suggests that the diversity of *Trichoderma* in this agroecosystem has been underestimated and remains largely uncharacterized. In this sense, the use of endophytic *Trichoderma* strains in the cacao production system could help reduce the incidence of economically important diseases, such as those caused by *Fusarium* spp. during the nursery stage and by *Moniliophthora roreri* in the productive phases.

Several studies have documented the ability of *Trichoderma* species to colonize plant tissues and activate mechanisms of induced resistance in the host plant, providing protection against biotic stresses (Tyśkiewicz et al., 2022; Guzmán-Guzmán et al., 2025; Yang et al., 2025). This ability, together with direct biocontrol mechanisms such as antibiosis and mycoparasitism, positions *Trichoderma* as a sustainable and promising alternative for the biological management of diseases in cacao systems (Leiva et al., 2020, 2022; Santillan-Culquimboz et al., 2025).

Antibiosis in *Trichoderma* involves the synthesis of secondary metabolites with antagonistic activity that inhibit the growth of pathogens (Santillan-Culquimboz et al., 2025), the effectiveness of which depends on the specific strain-pathogen interaction. This variability, attributed to genetic factors and pathogen resistance (Yao et al., 2023; Garcés-Moncayo et al., 2025), was evident in the HWSC19 and HWSC27 strains, which exhibited higher levels of inhibition against *M. roreri* than against *Fusarium* sp. In terms of overall performance, the inhibition observed against *M. roreri* (~60%) slightly surpassed the 55% reported by Reyes-Figueroa et al. (2016) and Leiva et al. (2020). In contrast, the activity against *Fusarium* sp. (~40%) was lower than the 60% described in previous literature (Chen et al., 2021; Modrzewska et al., 2022), corroborating the specificity of the antagonistic mechanism.

Mycoparasitism is a direct antagonism mechanism where *Trichoderma* degrades the cell wall of phytopathogenic hyphae through recognition, adhesion, and secretion of lytic enzymes (Mukherjee et al., 2021; Santillan-Culquimboz et al., 2025; Awal et al., 2024). The effectiveness of this process also proved to be host-specific, in agreement with previous studies (Leiva et al., 2020; Reyes-Figueroa et al., 2016; Garcés-Moncayo et al., 2025; Bailey et al., 2008). The HWSC21 and HWSC26 strains showed a significant differential efficacy (80% against *Fusarium* sp. vs. <15% against *M. roreri*). Although some strains were pathogen-dependent, strains with mycoparasitism levels exceeding 90% against the evaluated pathogens were also found, results that are consistent with those reported by Reyes-Figueroa et al. (2016) and Leiva et al. (2020).

The Potential Antagonism (PA) used in this study reflects the synergistic nature of the *Trichoderma*-pathogen interaction. As noted by Leiva et al. (2020) and Mukherjee et al. (2021), strains that combine mycoparasitism and antibiosis tend to show greater persistence and effectiveness in disease control, as they attack the pathogen through multiple mechanisms of action. A high PA value indicates that the strain not only competes for space and nutrients but also possesses the enzymatic and metabolic machinery to actively suppress the phytopathogen, which is a desirable criterion for selecting biocontrol agent candidates (Santillan-Culquimboz et al., 2025).

In this context, the strains related to *Trichoderma* cf. *sexdentis* (Viride clade) and *T. erinaceum* (HWSC27) showed the highest levels of antagonism, achieving nearly 80% suppression against *Moniliophthora roreri* and 70% against *Fusarium* sp. These results exceed those reported by Leiva et al. (2020) and are above the average observed in *Trichoderma* strains associated with cacao, which typically ranges around 60% (Leiva et al., 2022). While these *in vitro* results position the strains as promising candidates, field studies are necessary to validate their effectiveness under real conditions and their commercial viability (Santillan-Culquimboz et al., 2025).

## 5 Conclusion

This study demonstrates that the endophytic *Trichoderma* associated with *Theobroma cacao* in Peru presents a greater taxonomic diversity than previously documented. Through an integrative approach combining multilocus phylogenies and morphological characterization, both described species and phylogenetically differentiated lineages were identified, whose specific delimitation could not be fully resolved, suggesting the presence of cryptic diversity within the *Trichoderma* genus. This work also constitutes the first report of *Trichoderma sexdentis* and *T. strigosum* as endophytes of cacao in Peru, highlighting the limited previous exploration of endophytic communities in Peruvian cacao agroecosystems. From a functional perspective, several endophytic strains showed high antagonistic potential against *Moniliophthora roreri* and *Fusarium* sp., with isolates related to *Trichoderma cf. sexdentis* showing antagonism levels that equal or exceed those previously reported. Furthermore, the strain associated with *T. erinaceum* showed superior antagonism against *M. roreri*, while *T. sexdentis* demonstrated high potential against *Fusarium* sp., suggesting its potential application in nurseries and fields for pathogen control in cacao cultivation. These findings provide native biological alternatives for the sustainability of the crop and reduce dependence on chemical fungicides. The efficacy observed *in vitro* justifies conducting *in vivo* trials to validate performance under field conditions.

## Data Availability

The datasets presented in this study are available in online repositories. The analysis data are deposited in the GitHub repository (https://github.com/Henry-WSC/CacaoBioShield-Trichoderma), while the sequence data are deposited in GenBank (NCBI), and the corresponding accession numbers are provided in Table 1 of the article.
